# Post-treatment infection prediction in CLL using domain adaptation of lymphoma electronic health records

**DOI:** 10.2340/1651-226X.2026.44569

**Published:** 2026-02-19

**Authors:** Mehdi Parviz, Christian Brieghel, Mikkel Werling, Thomas Lacoppidan, Emelie Rotbain, Carsten U. Niemann, Rudi Agius

**Affiliations:** a Department of Biology, University of Copenhagen, Copenhagen, Denmark; b Department of Hematology, Copenhagen University Hospital, Rigshospitalet, Copenhagen, Denmark; c Danish Cancer Institute, Danish Cancer Society, Copenhagen, Denmark; d Department of Clinical Medicine, University of Copenhagen, Copenhagen, Denmark

**Keywords:** CLL, adverse events, infection, treatment, prediction, machine learning

## Abstract

**Background and purpose:**

Infections are the leading cause of morbidity and mortality in patients with chronic lymphocytic leukemia (CLL) and occur during and after treatment. When deciding on the type of CLL treatment, the risk of infections is typically assessed based only on age and comorbidities; therefore, there is a need to develop a predictive model that incorporates information from multiple data sources. However, training an effective machine learning model requires a large sample size.

**Patient/material and methods:**

In this study, we developed a machine learning approach using domain adaptation (DA) to predict the risk of severe infection during treatment in patients with CLL. We implemented a DA strategy using lymphoma patient data and compared it with a domain-specific (DS) strategy across multiple models.

**Results:**

The DA strategy outperformed the DS strategy across all models, with an odds ratio of 4.43 for infection risk between high-risk and low-risk groups, compared with an odds ratio of 3.69 for the best DS model and 2.27 for the CLL-IPI alone. Explainability analysis identified predictive features for both the DA and DS models, including medication data and biochemistry tests. Specifically, C-reactive protein levels and non-therapeutic drugs were common features identified by both DA and DS models, while the DA models relied more heavily on alimentary tract drugs, solvents and diluting agents, and antibacterial medications.

**Interpretation:**

These findings highlight the value of integrating data from different diseases (lymphoma) to improve predictions in a target disease (CLL), and represent a step toward data-driven identification of CLL patients at high risk of infection during treatment.

## Graphical abstract

**Figure:**
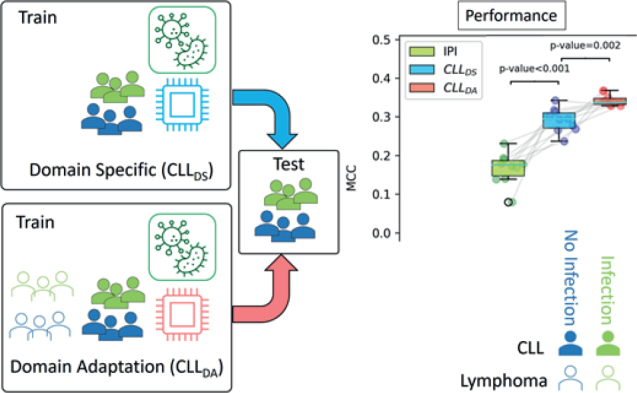

## Introduction

Assessing infection risk upon treatment for chronic lymphocytic leukemia (CLL) is essential, as common treatments such as chemoimmunotherapy (CIT) and targeted therapies can weaken the immune system [1, 2]. Recent studies highlight the complex interactions between the development and growth of CLL cells, and the targeted therapies [3, 4]. By impairing immune function, CIT and targeted treatments increase susceptibility to infections [5, 6], leading to reduced quality of life [7] and greater healthcare costs [8]. As CLL treatments have advanced considerably over the last decade, evaluating the risk of adverse events, especially infections, remains crucial. Treatment should ideally be tailored to each patient’s condition within a personalized medicine approach.

Developing a scoring or prediction model can help doctors to more easily identify high-risk patients. Such models have proven to be effective in various clinical contexts, including emergency medicine [9], pediatrics [10], acute myeloid leukemia [11], CLL [12], solid cancers [13], and infectious diseases [14]. Previous studies have examined whether machine learning (ML) can predict infection risk at the time of diagnosis [15, 16]. Recently, an ML-based model designed to predict the combined risk of treatment and infection (also known as CLL-TIM), has been integrated into a hospital information system [17]. This model demonstrated that incorporating a larger volume of data, including more variables and long-term patient histories, along with advanced ML algorithms, leads to improved predictive performance compared to traditional scoring systems. While the CLL-TIM model has been successful in predicting the combined risk of treatment and infection at the time of diagnosis, its utility during the treatment phase is limited. This is because it was not trained on post-diagnosis data and does not account for information related to treatment types. Moreover, CLL-TIM has shown lower accuracy in predicting infections compared to treatment-related risks. Given the challenges of infection prediction and the evidence that historical data combined with ML can improve accuracy, we explored whether incorporating data from other diseases can further enhance infection prediction for CLL patients during treatment.

However, achieving strong performance with ML models usually requires large sample sizes. In medical domain, assembling such extensive datasets is difficult due to strict privacy regulations and the high cost of generating clinical data, such as diagnostic tests or sequencing [18, 19]. This issue limits the performance of domain-specific (DS) models specially when dealing with rare diseases [20]. In machine learning, the challenge of small sample sizes is often addressed with Domain Adaptation (DA), which augments target data with data from related domains (e.g., diseases with similar traits) [20, 21]. DA and, more broadly, transfer learning offer practical, theoretically grounded ways to leverage larger cancer datasets to improve prediction in a narrower target domain. DA assumes identical feature spaces and tasks, which applies here because all domains used the same features and outcome definition [20].

Although leukemia types differ biologically in tissue origin, mutations, and progression, transfer learning remains justified for several reasons. Firstly, ML models trained on large cohorts often learn generalizable features, such as age, blood markers, and medication use, that are common across hematologic malignancies. Such models provide a strong starting point even when fine-tuned on limited data from a specific cancer.

Secondly, annotated outcome data for rare cancer types are scarce, making training complex models from scratch both impractical and prone to overfitting. Transfer learning leverages the statistical richness of large, diverse datasets and introduces inductive biases that stabilize learning in low-data settings [20].

Thirdly, modern methods such as DA and selective fine-tuning mitigate domain mismatch by allowing models to adapt to the biological and clinical specifics of the target disease while retaining useful shared representations.

Finally, empirical studies consistently show performance gains when pre-trained medical models are adapted to specific cancers for tasks such as survival prediction [12], disease subtyping [22], and mortality prediction [23]. These findings indicate that, despite biological differences, clinical patterns overlap sufficiently for transfer learning to be both viable and beneficial.

Overall, transferring knowledge from broad cancer datasets to specific cancer types is often essential for accurate clinical outcome prediction, balancing data scarcity with biological variability.

In this study, we proposed a DA strategy using lymphoma data to train binary classifiers for predicting severe infection risk in treated CLL patients, with DS models as baselines. For DA, we used Balanced Weighting, a model-agnostic method compatible with various ML models [24]. We also employed explainable AI techniques to identify the most influential predictive features [25]. To our knowledge, this is the demonstration of an ML–based approach to infection prediction that improves performance by incorporating data from other leukemia types. This study establishes the feasibility of DA for infection prediction and opens avenues for future research, including models for other adverse events and continuous, time-aware risk prediction beyond diagnosis or treatment time.

## Patients/material and methods

### Data sources and patients

We assembled a cohort of patients with CLL (Table 1) and lymphomas (Table S1) receiving first-line treatment from the launch of the EPIC^®^-based Electronic Health Record (EHR) system in the Eastern Denmark (known as Sundhedsplatformen [SP]) between May 2016 and August 2023. Here, small lymphocytic lymphoma (SLL) is considered the same biological entity as CLL. Patients with CLL and B-cell lymphoma from eastern Denmark (i.e., the Capital Region and Region Zealand) were identified from the Danish Clinical Quality Program – National Clinical Registries (RKKP) diagnosed since 2008 in the Danish National Chronic Lymphocytic Leukemia Registry (DCLLR) and since 2005 the Danish National Lymphoma Registry (LYFO), respectively [26, 27]. All data were retrieved and analyzed using the DALY-CARE data resource (Supplementary Data sources) [28].

**Table 1:** Biological and clinical characteristics of CLL patients grouped by treatment category.

	FCR or BR	CD20Clb or Clb	Venetoclax-based therapy^a^	Ibrutinib monotherapy	Other^b^	All patients
Number of patients	113	53	69	29	37	301
Age (median (IQR))	66 (60–71)	79 (73–83)	62 (55–68)	66 (58–74)	73 (66–78)	68 (60–75)
Sex, male	81 (72)	35 (66)	53 (77)	19 (66)	16 (43)	204 (68)
Binet stage at diagnosis						
A	87 (77)	40 (75)	50 (72)	20 (69)	29 (78)	226 (75)
B	16 (14)	8 (15)	13 (19)	6 (21)	5 (14)	48 (16)
C	10 (9)	5 (9)	6 (9)	3 (10)	3 (8)	27 (9)
B2M at diagnosis						
≤4.0 mg/L	78 (69)	25 (47)	57 (83)	17 (59)	25 (68)	202 (67)
>4.0 mg/L	20 (18)	26 (49)	11 (16)	11 (38)	6 (16)	74 (25)
Missing	15 (13)	2 (4)	1 (1)	1 (3)	6 (16)	25 (8)
IGHV status						
Mutated	45 (40)	15 (28)	16 (23)	4 (14)	13 (35)	93 (31)
Unmutated	50 (44)	21 (40)	38 (55)	21 (72)	13 (35)	143 (48)
Unknown	18 (16)	17 (32)	15 (22)	4 (14)	11 (30)	65 (22)
*TP53*						
Mutated	2 (2)	1 (2)	4 (6)	3 (10)	4 (11)	14 (5)
Wild type	40 (35)	17 (32)	23 (33)	8 (28)	9 (24)	97 (32)
Missing	71 (63)	35 (66)	42 (61)	18 (62)	24 (65)	190 (63)
FISH status						
del(17p)	1 (1)	1 (2)	4 (6)	9 (31)	7 (19)	22 (7)
del(11q)	19 (17)	9 (17)	9 (13)	2 (7)	1 (3)	40 (13)
tri(12)	22 (19)	11 (21)	8 (12)	2 (7)	4 (11)	47 (16)
Normal	12 (11)	4 (8)	10 (14)	5 (17)	1 (3)	32 (11)
del(13q)	43 (38)	13 (25)	23 (33)	9 (31)	15 (41)	103 (34)
Unknown	16 (14)	15 (28)	15 (22)	2 (7)	9 (24)	57 (19)
Performance status at diagnosis						
0	99 (88)	41 (77)	63 (91)	25 (86)	35 (95)	263 (87)
1	12 (11)	9 (17)	6 (9)	3 (10)	2 (5)	32 (11)
>1	2 (2)	3 (6)	0 (0)	1 (3)	0 (0)	6 (2)
CLL-IPI at diagnosis						
Very high	0 (0)	0 (0)	2 (3)	4 (14)	2 (5)	8 (3)
High	13 (12)	9 (17)	6 (9)	7 (24)	7 (19)	42 (14)
Intermediate	29 (26)	15 (28)	24 (35)	12 (41)	5 (14)	85 (28)
Low	31 (27)	6 (11)	9 (13)	0 (0)	7 (19)	53 (18)
Unknown	40 (35)	23 (43)	28 (41)	6 (21)	16 (43)	113 (38)

Note. *n* (%) unless otherwise specified.

a Venetoclax monotherapy or in combination with any of the following treatments: ibrutinib or other BTK inhibitors, rituximab, or obinutuzumab.

b Other treatments such as ABVD, CHOEP, rituximab monotherapy, and bone marrow transplantation were included in the category. B2M: β2-microglobulin.

### Infection definition

Severe infection was defined as being administered IV antimicrobials and having a blood culture drawn concurrently (within two days of each other) in a 1-year post-treatment initiation outlook. Only the intravenous antimicrobials defined by a set of ATC codes (J01-J05 and P01) were included; oral antimicrobials and prophylactic antimicrobials (such as sulfamethoxazole with trimethoprim [J01EE01] and acyclovir/valacyclovir [J05AB01/J05AB11]) were excluded. To exclude cases where antimicrobials were administered merely on suspicion of infection, we only included antimicrobial treatments with a duration of more than 24 h.

### Data preprocessing and feature extraction

Hospitalization history was defined as being hospitalized for more than 24 h within one year prior to treatment. In a few cases, patients underwent treatment while already experiencing severe infections. These cases, which were categorized as continuation of infection, were excluded from the test set to avoid bias, i.e., predicting obvious instances. Patients who died or had a follow-up period of less than a year after the treatment, and who did not experience a severe infection, were also excluded.

Prediction point was defined at the time of first-line treatment. To avoid future information leakage only data available prior to the prediction point were used for feature extraction and aggregation.

To make the collected data usable by a ML algorithm, we performed data preprocessing and feature extraction steps customized for a group of variables as detailed in the following. Binary variables such as sex, B2M, FISH, IGHV and *TP53* status, were directly encoded as features. Categorical variables, like treatment types, with *L* levels were converted into *L* binary features indicating the presence of the corresponding level.

Feature extraction from timeseries data was performed using the timeseriesflattener Python package [29]. Three lookback periods (30, 90 and 365 days), ending at the prediction point, were defined to aggregate variable values using several aggregation functions. Previous ICD-10 diagnoses and SNOMED codes, as well as prescribed and administered medications, were aggregated using counts. For prescribed and administered medications, the cumulative dosage of individual drugs was also calculated. From microbiology cultures and analyses, the total number of events including drawn blood cultures were extracted. In previous studies, drawn blood cultures has been used as a proxy for clinical infection [16, 30, 31]. For biochemistry tests, multiple aggregators such as the minimum, maximum, average, number of tests, and the most recent value prior to the prediction point were employed, for example, low IgG, IgM, and IgA levels were extracted using this approach. The missing values of numerical features were imputed using the median value estimated during training. Features that had many missing values (more than 90%) were removed from the data. In total, 5,749 features were extracted, of which the complete list is provided in the Supplementary materials.

### Modeling and statistical analysis

**Figure 1:**
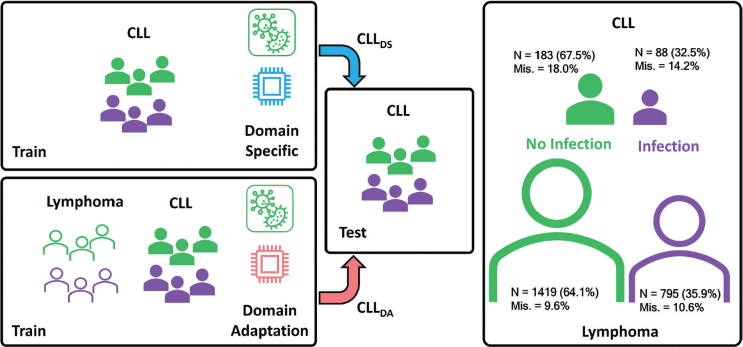
Schematic representation of the domain-specific and proposed domain adaptation strategies for training a predictive model of infection in CLL. Domain-specific (DS) strategy uses only CLL samples for training, while domain adaptation (DA) strategy exploits extra samples from lymphoma patients. Both strategies are evaluated on CLL sample only. The number of patients and the proportion of each group, infection and no infection, as well as missing rate are provided.

In this study, we framed severe infection prediction as a binary classification problem. Three strategies were advised to investigate the benefit of a DA compared to a DS modeling and disease-specific International Prognostic Score (IPI) [32]. In the DS strategy (CLL_DS_), only data from CLL patients were used to develop classifiers, while in the DA strategy (CLL_DA_) data from patients with lymphoma were also included, but only during the training (Figure 1). The third strategy limited the features included in the CLL-IPI index [32]. Three classifiers including Logistic Regression (LR), Light Gradient-Boosting Machine (LGBM), and Radom Forest (RF), were developed using the three strategies.

We opted to use Balanced Weighting [24], a model agnostic technique, which allows for optimizing the ratio by which the samples from the source and the target domain contribute during training. We used ADAPT Python package to implement Balanced Weighting [33].

We next evaluated the models using stratified K-Fold cross-validation with K set to five. We repeated the K-Fold 10 times by introducing different data splits to measure the variability of the performance and subsequently a more robust estimate of the feature contributions. The contribution of the features and their interactions were measured using SHapley Additive exPlanations (SHAP) [25]. The preprocess and data compilation steps were performed in R, whereas the modeling and post analysis codes were developed in Python. Several Python packages including Scikit-learn [34], Scikit-survival [35], and lifelines [36] were used to perform feature extraction, K-fold cross-validation, and to generate cumulative incidence plots and statistical tests, respectively. The Matthews correlation coefficient (MCC) was used to evaluate the performance of the classifiers as it is a more reliable metric to measure the performance of a binary classifier compared with other metrics [37]. The significance of the differences between models’ performances were tested using paired t-tests. Overall survival (OS) was calculated by Kaplan–Meier estimator implemented by survival package in R [38]. Finally, we performed the proportional odds cumulative modeling to compute the odds ratio (OR) across risk groups for each strategy [39].

## Results

### Study population

The cohort consisted of 301 patients with CLL and 2,397 patients with lymphoma, who had received first-line treatment between May of 2016 and August of 2023. The inclusion criteria are depicted in a CONSORT diagram (Figure S1).

The median age at diagnosis was 68 years for CLL patients (interquartile range [IQR]: 60−75) and 68 years (IQR: 56−76) for lymphoma patients (Table S1). Patients with CLL were followed in active surveillance for a median of 31 months, whereas patients with non-Hodgkin lymphoma or other non-DLBCL lymphoma (*n* = 954) were followed for a median of one month before starting therapy (Table S1). The median age at the time of treatment was 72 years (IQR: 64−78) and 68 years (IQR: 56−76) for CLL and lymphoma, respectively. The median follow-up after treatment for CLL patients was 3.9 years (IQR: 2.5−5.1) and 3.1 years (IQR: 1.7−4.7) for patients with lymphoma. The 1-year OS from first-line treatment was 87% (95% confidence interval [CI]: 84%−91%) for CLL and 89% (CI: 88%−90%) for lymphoma. Considering death as a competing risk, the 1-year cumulative incidence rate of severe infections after treatment among CLL patients was 35.5% and 33.3% among lymphoma patients.

Of the 301 patients with CLL, 290 (96.3%) had either an infectious event or a full one-year follow-up. Those who did not have a full one-year follow-up were included in the survival time analysis. Of the remaining patients, 19 had an infection close to the time of treatment and were therefore not included during testing but were used for training. The prevalence of severe infection among the patients used in testing was 32.5% (*n* = 88/271). Out of 2,397 patients in the lymphoma cohort, 2,214 (92.4%) had an infectious event or a full-one year follow-up. Since lymphoma patients were excluded from the test set, those with infections near treatment were included in training. The prevalence of severe infection among the lymphoma patients used in training was 35.9% (*n* = 795/2,214).

### Lymphoma data improve the predictions for CLL patients through domain adaptation

**Figure 2:**
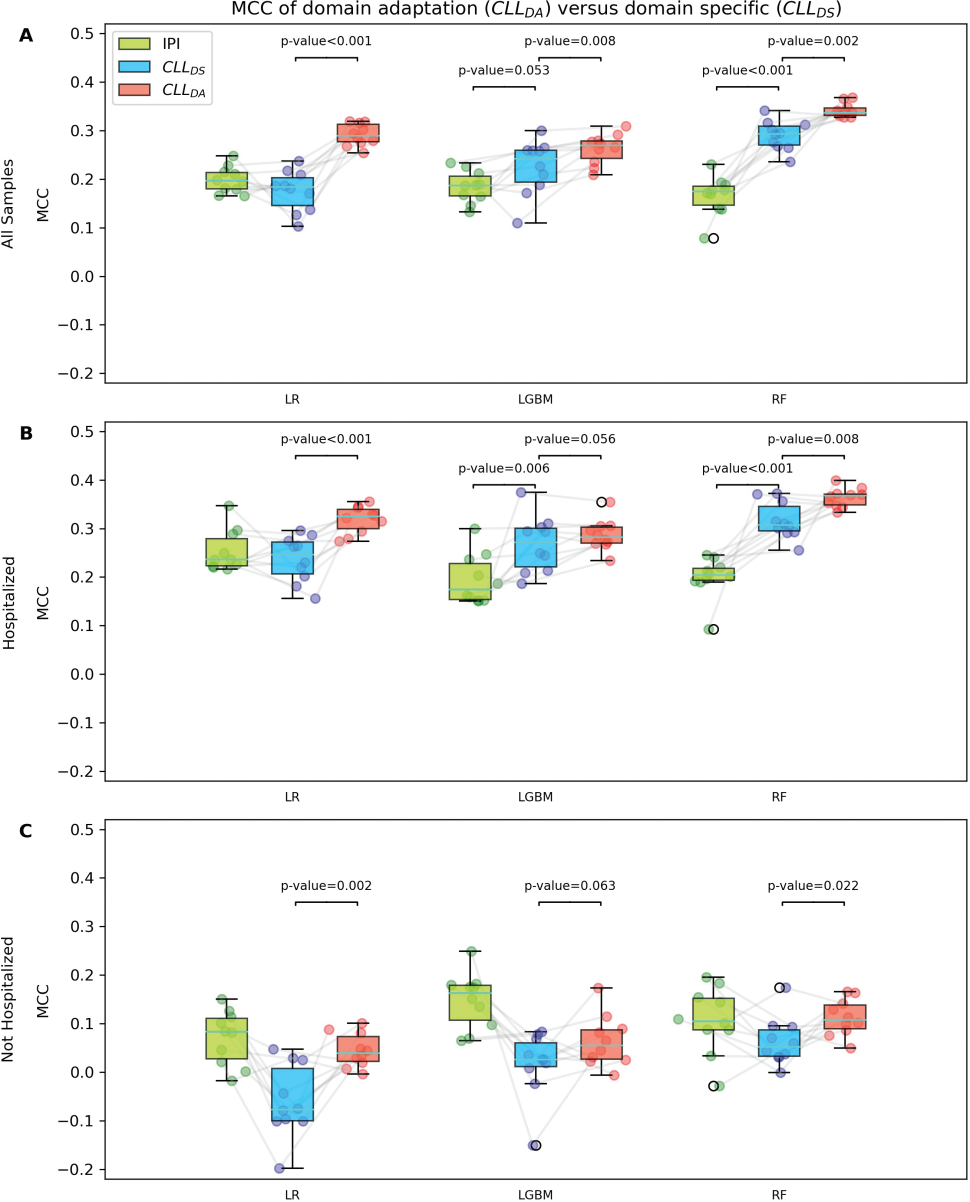
Performance measured by MCC of infection prediction strategies using different models. Box plot of IPI the domain-specific, and the domain adaptation strategies across three models, i.e., Logistic Regression (LR), Light Gradient-Boosting Machine (LGBM), and Radom Forest (RF) on all samples (A), patients with hospitalization history (B), and patients without hospitalization history (C). The significance tests were performed using paired t-test. MCC: Matthew’s correlation coefficient.

We compared the performance of the three strategies, i.e., IPI, CLL_DS_, and CLL_DA_, using three models, namely LR, LGBM, and RF. Measured by MCC, CLL_DA_ outperformed both CLL_DS_ and IPI across the three models and CLL_DS_ ranked higher than IPI in two (RF and LGBM) out of the three models (Figure 2A). Among the three models, RF achieved the best performance across all the three strategies with an MCC of 0.166 (0.126–0.206), 0.290 (CI: 0.255–325) and 0.342 (CI: 0.309–0.375) for IPI, CLL_DS_, and CLL_DA_, respectively.

**Figure 3:**
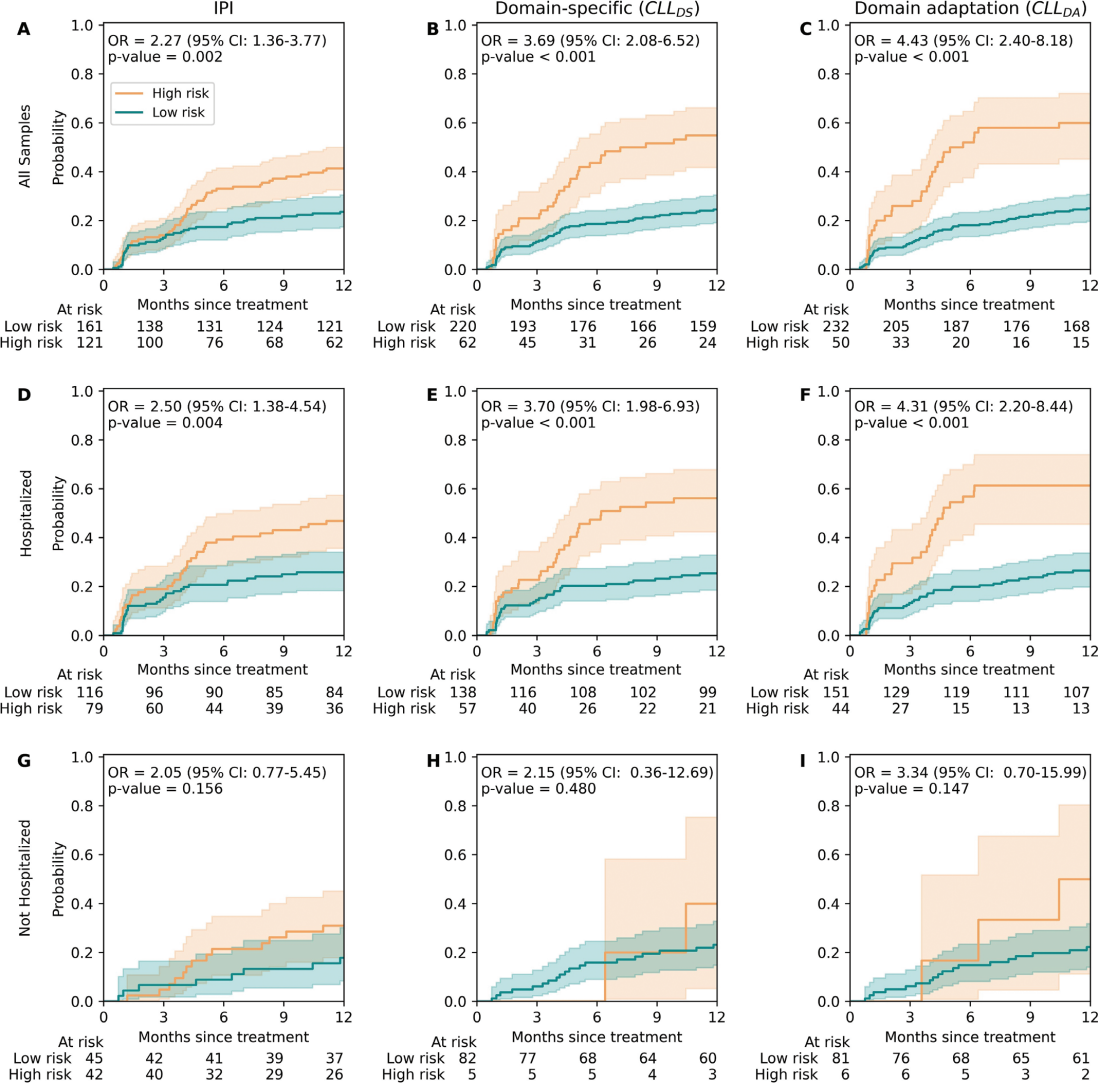
Cumulative incidence curve of time-to-first infection estimated from the prediction point on all samples (A, B, C), patients with hospitalization history (D, E, F), and patients without hospitalization history (G, H, I) using IPI model (A, D, G), DS strategy (B, E, H), and DA strategy (C, F, I). CI: 95% confidence interval. P-values were calculated by Gray’s test.

We further investigated the RF model’s performance across the three strategies using cumulative incidence plots of the samples classified by the model as either high risk or low risk. When comparing the results across all samples, we observed that CLL_DA_ showed better separation between high-risk and low-risk cases compared with CLL_DS_ and IPI (Figure 3A-C). The confusion matrices for the IPI, CLL_DS_, and CLL_DA_ strategies using RF, the best-performing model evaluated on all patients, are shown in Table S2. Overall, the confusion matrices indicate that CLL_DA_ achieved a substantially lower false acceptance rate and higher precision, but at the cost of a higher false rejection rate and lower recall, compared with IPI and CLL_DS_.

### Infection predictions are more accurate for patients with a hospitalization history

**Figure 4:**
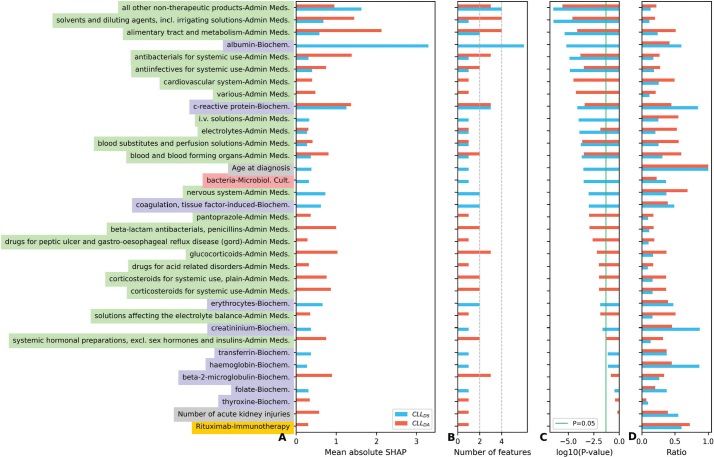
Most informative feature groups predictive of infection. Accumulated absolute SHAP values of all features constructed from each variable, one variable can be represented by several features as detailed in methods, (B) alongside the number of features from each variable (A) for both domain-specific and domain adaptation strategies. Univariate Mann–Whitney U test was performed for ranking of feature groups based on the average P-value (C). The ratio of samples with non-missing values (D). Feature names are color coded: Medications (green), Biochemistry (purple), Treatment (orange), and others in grey.

As a significant proportion of the identified high ranked features (Figure 4A) upon DA was derived from variables recorded during hospitalization for a certain period (at least 24 hours), we specifically assessed whether the performance of the RF model was sensitive to prior hospitalizations. To achieve this, we stratified patients based on hospitalization history. The majority of the CLL and lymphoma patients had a history of hospitalization (69.0% or 187 and 69.2% or 1,531, respectively). The rate of severe infection in a 1-year outlook post-treatment for CLL patients with and without a hospitalization history was 35.8% (*n* = 67) and 25.0% (*n* = 21), respectively. Likewise, the proportion of lymphoma patients with and without hospitalization was 41.5% (*n* = 636) and 23.3% (*n* = 159), respectively.

We investigated the effect of prior hospitalization on the results by evaluating all the nine models stratified on hospitalization history. When testing on samples with a hospitalization history, RF achieved the best performance across all the three strategies with MCC of 0.361 (CI: 0.319–0.404), 0.320 (CI: 0.280–0.360) and 0.200 (0.149–0.251) for CLL_DA_, CLL_DS_, and IPI, respectively (Figure 2B). All three strategies showed improved MCCs relative to the results obtained on the full patient cohort (Figure 2A vs. 2B).

For patients without a hospitalization history, the models underperformed compared to those with a hospitalization history across all three strategies. For comparison, in the case of RF, the MCCs dropped to 0.113 (CI: 0.052–0.174), 0.075 (CI: 0.015–0.134) and 0.115 (0.036–0.195) for CLL_DA_, CLL_DS_, and IPI, respectively (Figure 2C).

### Improving survival time distribution stratification by DA

The analysis of cumulative incidence using the proportional odds model also showed that employing DA strategy improved the odd ratio (OR) (Figure 3A–C). Similar analysis also showed a better performance of the models on patients with hospitalization history (Figure 3D–F) compared to patients without hospitalization history (Figure 3G–I).

### Administered medications and biochemistry tests predict severe infection

To identify the features contributing most to these models, we performed explainability analysis. The RF model trained using CLL_DA_ was mainly using features extracted from administered medications including non-therapeutic drugs (ATC V07, e.g., disinfectants and ban-aids), alimentary tract drugs (ATC A, e.g., proton pump inhibitors and antiemetics), solvent and diluting agents, antibacterial medications, and biochemistry results including c-reactive protein level (Figure 4A). Out of 46 features, 37 (~80%) features were extracted from administered medications and eight were extracted from biochemistry tests (Figure 4B). In case of CLL_DS_, an equal number of features were identified from biochemistry tests and administered medications at 16 (~47%) out of 34, favoring biochemistry test with higher contributions (Figure S1). The most important features were albumin and C-reactive protein level extracted from biochemistry tests and non-therapeutic drugs from administered medications. The number of previously drawn blood cultures and age were among the predictive features, but only for DS. Overall, DA resulted in a larger number of features, (46 versus 34 with no DA) that according to Mann–Whitney U test majority of them were statistically significant (Figure 4C).

## Discussion and conclusion

Infections are a major concern for CLL patients, impacting treatment and quality of life. For medically frail patients or those with comorbidities, infections can be life-threatening. Identifying high-risk patients may help physicians to select optimal treatments. Building on previous studies [15, 16], we aimed to predict severe infection risk upon treatment in a CLL population in Eastern Denmark, using first-line treatment data. Given the low number of CLL cases, we explored whether data from neighboring diseases, such as lymphomas, could improve predictive performance through DA.

Our results showed that including lymphoma patient data in the CLL training pool improved infection predictions for CLL patients. Compared with training on CLL data alone, DA increased the number of predictive features for infections occurring during first-line treatment. This suggests that lymphoma data may help to identify infection risk factors in CLL. Although DA and transfer learning are common in other fields, their use in healthcare has largely been limited to medical imaging [40] due to challenges in integrating diverse electronic health records [41]. Access to the DALY-CARE data resource helped overcome these challenges by enabling cross-disease analysis [28]. While CLL and lymphomas differ in some characteristics, their clinical management is often similar, as in cases of febrile neutropenia.

Our analysis showed that most patients in both the CLL and lymphoma cohorts had been hospitalized prior to initiating treatment. Because the risk factors predictive of severe infection during treatment were derived primarily from hospital-based data, the models achieved higher accuracy for patients with a prior hospitalization history and lower accuracy for those without one. This underscores the challenge of predicting infections in patients without previous hospitalizations, likely reflecting their generally better health status and lower baseline risk of severe infection, as well as the potential influence of surveillance bias.

The explainability analysis of DS models confirmed previous findings that biochemistry test features are predictive [15, 16], while the DA strategy identified additional features related to patient comorbidity and frailty prior to treatment. This reinforces the link between comorbidities and increased risk of infection and mortality [42]. Antibacterial, anti-infective, and immunosuppressive medications (e.g., glucocorticoids and corticosteroids) were predictive of severe infection, consistent with prior findings associating pre-diagnosis antibiotic use with worse outcomes in CLL [43]. The association between alimentary tract medication and infection risk may be indirect, as it includes diverse medications like proton-pump inhibitors and cardiovascular drugs [44, 45]. However, the CLL comorbidity index highlighted gastrointestinal, metabolic, and cardiovascular comorbidities as key prognostic indicators in CLL and lymphoma [46, 47]. C-reactive protein, identified by both DS and DA, may reflect prior infections or disease-driven reactive states [48]. Albumin levels, a strong predictor by DS, are often monitored for kidney, liver, and nutritional status, with low levels suggesting frailty and heightened infection risk [49].

The results are comparable to our previous study with a similar sample size, which assessed risk from time of CLL diagnosis rather than from time of treatment [15]. Analyzing patients based on hospitalization history showed that both DS and DA strategies, and to a lesser extent CLL-IPI, had better predictive performance for recently hospitalized patients. Likely, hospitalization history provided more details about patient comorbidities, yielding more informative features that enhanced prediction [46].

Our analysis is limited to using lymphoma patients to test DA on CLL patients. Future work should focus on extending our DA approach by including data from other hematological malignancies such as multiple myeloma might be beneficial for DA. Including treatment naïve CLL patients as source data also might improve the performance of DA strategy. Predicting less severe infection cases as an auxiliary outcome could help conditions that are precursor of severe cases. In addition, this probably improves the performance for patients that have no hospitalization history. From a modeling perspective, employing DA techniques that learn the shifts in data across diseases [20], along with the use of deep learning models, may be a promising approach to explore.

## Supplementary Material



## Data Availability

Under Danish and EU legislation, the data cannot be deposited in a public repository; however, it can be accessed through DALYCARE upon reasonable request.
