# Supplementary material for: Post-treatment infection prediction in CLL using domain adaptation of lymphoma electronic health records
Source: Acta Oncol. 2026 Feb 19;65:44569. doi: 10.2340/1651-226X.2026.44569 (PMC12927150; doi:10.2340/1651-226X.2026.44569)
Supplement: Supplementary file 1 [file AO-65-44569-s1.pdf]

Supplementary material has been published as submitted. It has not been copyedited, or typeset by Acta Oncologica

Supplementary Information

Post-Treatment Infection Prediction in CLL Using Domain Adaptation of Lymphoma Electronic Health Records

Materials and Methods

I. Data sources

The date of first-line treatment and the type of treatment were extracted from the RKKP. The date of drawn blood cultures was extracted from the Clinical Laboratory Information System Database with routine laboratory results (LABKA).[1] The data on intravenous (IV) antimicrobial usage were obtained from the administered medicines module in SP. Preprocessed LABKA data including biochemistry results, all microbiology cultures and analyses were retrieved from the Personalized Medicine of Infectious Complication in Immune Deficiency (PERSIMUNE) data warehouse.[2]

Baseline characteristics at diagnosis such as such as biological sex, age, Binet stage, fluorescence in situ hybridization (FISH) abnormalities, immunoglobulin heavy-chain variable (IGHV) mutational status, β-2 microglobulin (B2M), and *TP53* status were obtained from the DCLLR, and CLL international prognostic index (CLL-IPI) was calculated as previously described.[3]

Prescribed medications were extracted from the Register of Pharmaceutical Sales (LSR), and SNOMED codes were retrieved from the Danish National Pathology Register.[4,5] Previous diagnoses were retrieved from three sources, i.e., the Danish National Patient Registry (LPR)[5] versions 1 and 3, admissions modules in SP, and RKKP for CLL/lymphoma diagnoses.[3,6] Diagnoses were encoded using the 10th revision of International Classification of Diseases (ICD-10. Administered medications were compiled by combining the National Hospital Medication Register (SMR) and the administered medicines module in SP.[7] Anatomical Therapeutic Chemical (ATC) codes was used to classify medications. Hospitalization history prior to treatment was extracted by merging SP and LPR admission data sources.

II. Ethical approvements

For this study, the required ethical approvals were obtained from appropriate authorities, including the Danish Health and Medicine Authorities (jr. no. 3-3013-1141/1), Danish National Ethics Committee (1804410), and the Danish Data Protection Agency (jr. no. RH-2015-96 03856).

Results

I. Lymphoma Population

Table S1. Biological and clinical characteristics of Lymphoma patients grouped by treatment category.

Covariate	RCHOP	Rituximab	BR	ABVD	Other	All patients
**Number of patients**	907	565	326	267	332	2397
**Age (median (IQR))**	71 (65-76)	65 (55-75)	70 (60-76)	41 (27-60)	69 (53-77)	68 (56-76)
**Sex, male**	507 (56)	334 (59)	184 (56)	143 (54)	179 (54)	1347 (56)
**Performance status at diagnosis**						
**0**	572 (63)	340 (60)	225 (69)	236 (88)	250 (75)	1623 (68)
**1**	223 (25)	138 (24)	82 (25)	24 (9)	48 (14)	515 (21)
**>1**	112 (12)	87 (15)	19 (6)	7 (3)	34 (10)	259 (11)
**Stage**						

**1**	169 (19)	87 (15)	12 (4)	29 (11)	180 (54)	477 (20)
**2**	142 (16)	40 (7)	21 (6)	110 (41)	52 (16)	365 (15)
**3**	147 (16)	67 (12)	37 (11)	75 (28)	20 (6)	364 (14)
**4**	449 (50)	365 (65)	255 (78)	53 (20)	78 (23)	1200 (50)
**Unknown**	0 (0)	6 (1)	1 (0)	0 (0)	2 (1)	9 (0)
**B symptoms**	318 (35)	201 (36)	107 (33)	109 (41)	63 (19)	798 (33)
**No. of extranodal sites**						
**0**	281 (31)	90 (16)	58 (18)	204 (76)	132 (40)	765 (32)
**1**	331 (36)	301 (53)	185 (57)	45 (17)	151 (45)	1013 (42)
**>1**	295 (33)	174 (31)	83 (25)	18 (7)	49 (15)	619 (26)
**Bulky tumor**						
**(>10 cm)**	128 (14)	84 (15)	46 (14)	22 (8)	15 (5)	295 (12)
**Unknown**	148 (16)	172 (30)	80 (25)	26 (10)	94 (28)	520 (22)
**Bone marrow involvement**						
**0**	626 (69)	280 (50)	91 (28)	191 (72)	254 (77)	1442 (60)
**1**	200 (22)	235 (42)	221 (68)	56 (21)	61 (18)	773 (32)
**Unknown**	81 (9)	50 (9)	14 (4)	20 (7)	17 (5)	182 (8)
**Elevated lactate dehydrogenase**	435 (48)	270 (48)	109 (33)	105 (39)	85 (26)	1004 (42)
**Histologic findings**						
**cHL[a]**	11 (1)	11 (2)	0 (0)	260 (97)	63 (19)	345 (14)
**DLBCL[b]**	751 (83)	305 (54)	5 (2)	0 (0)	40 (12)	1101 (46)
**FL[c]**	83 (9)	37 (7)	117 (36)	0 (0)	88 (27)	325 (14)
**HD-LP[d]**	2 (0)	4 (1)	0 (0)	7 (3)	12 (4)	25 (1)
**MCL[e]**	27 (3)	59 (10)	50 (15)	0 (0)	4 (1)	140 (6)
**MZL[f]**	4 (0)	54 (10)	57 (17)	0 (0)	75 (23)	190 (8)
**NHL[g]**	23 (3)	35 (6)	9 (3)	0 (0)	25 (8)	92 (4)
**SLL[h]**	5 (1)	8 (1)	13 (4)	0 (0)	12 (4)	38 (2)
**WM[i]**	1 (0)	52 (9)	75 (23)	0 (0)	13 (4)	141 (6)
**IPI[j] at diagnosis**						
**0-1**	235 (26)	118 (21)	58 (18)	165 (62)	193 (58)	769 (32)
**2**	229 (25)	199 (35)	132 (40)	69 (26)	74 (22)	703 (29)
**3**	242 (27)	174 (31)	107 (33)	25 (9)	44 (13)	592 (25)
**4-5**	195 (21)	62 (11)	26 (8)	6 (2)	15 (5)	304 (13)
**Unknown**	6 (1)	12 (2)	3 (1)	2 (1)	6 (2)	29 (1)

Note. *n* (%) unless otherwise specified. [a]Classical Hodgkin Lymphoma (cHL), [b]Diffuse Large B-Cell Lymphoma (DLBCL), [c]Follicular Lymphoma (FL), [d]Lymphocyte-predominant Hodgkin lymphoma (HD-LP), [e]Mantle Cell Lymphoma (MCL), [f]Marginal Zone Lymphoma (MZL), [g]Non-Hodgkin Lymphoma (NHL), [h]Small Lymphocytic Lymphoma (SLL), [i]Waldenström Macroglobulinemi (WM), [j]IPI, Standard International Prognostic

II. Feature list

III. Confusion matrix

Table S2. Confusion matrices averaged across 10 runs of International Prognostic Index (IPI), domain-specific (DS), and domain adaptation (DA) strategies using RF when evaluated on all samples.

**IPI**	Prediction: No infection	Prediction: Infection	SD[a]
Label: No Infection	115.5[b] (63.1)	67.5 (36.9)	4.9

Label: Infection	40.7 (46.2)	47.3 (53.8)	1.6
**DS**	Prediction: No infection	Prediction: Infection	SD
Label: No Infection	158.2 (86.4)	24.8 (13.6)	2.5
Label: Infection	54.2 (61.6)	33.8 (38.4)	1.4
**DA**	Prediction: No infection	Prediction: Infection	SD
Label: No Infection	167.9 (91.7)	15.1 (8.3)	0.9
Label: Infection	56.9 (64.7)	31.1 (35.3)	1.1

If there is no description, count and (%) is provided. [a] Since in each row the sum is equal to the number of samples in each class (a constant), the standard deviations are equal, [b] The confusion matrix entries are averaged across multiple runs, resulting in float values.

Table S3. Confusion matrices averaged across 10 runs of International Prognostic Index (IPI), domain-specific (DS), and domain adaptation (DA) strategies using RF when evaluated on samples with hospitalization history.

**IPI – Hospitalized**	Prediction: No infection	Prediction: Infection	SD
Label: No Infection	81.5 (67.9)	38.5 (32.1)	3.9
Label: Infection	32.1 (47.9)	34.9 (52.1)	1.5
**DS – Hospitalized**	Prediction: No infection	Prediction: Infection	SD
Label: No Infection	98.5 (82.1)	21.5 (17.9)	2.2
Label: Infection	35.5 (53.0)	31.5 (47.0)	1.2
**DA – Hospitalized**	Prediction: No infection	Prediction: Infection	SD
Label: No Infection	106.8 (89.0)	13.2 (11.0)	0.8
Label: Infection	38.9 (58.1)	28.1 (41.9)	1.1

Table S4. Confusion matrices averaged across 10 runs of International Prognostic Index (IPI), domain-specific (DS), and domain adaptation (DA) strategies using RF when evaluated on samples without hospitalization history.

**IPI – Not hospitalized**	Prediction: No infection	Prediction: Infection	SD
Label: No Infection	34.0 (54.0)	29.0 (46.0)	1.9
Label: Infection	8.6 (41.0)	12.4 (59.0)	1.0
**DS – Not hospitalized**	Prediction: No infection	Prediction: Infection	SD
Label: No Infection	59.7 (94.8)	3.3 (5.2)	1.4
Label: Infection	18.7 (89.1)	2.3 (10.1)	0.5
**DA – Not hospitalized**	Prediction: No infection	Prediction: Infection	SD
Label: No Infection	61.1 (97.0)	1.9 (3.0)	0.3
Label: Infection	18.0 (85.7)	3.0 (14.3)	0.0

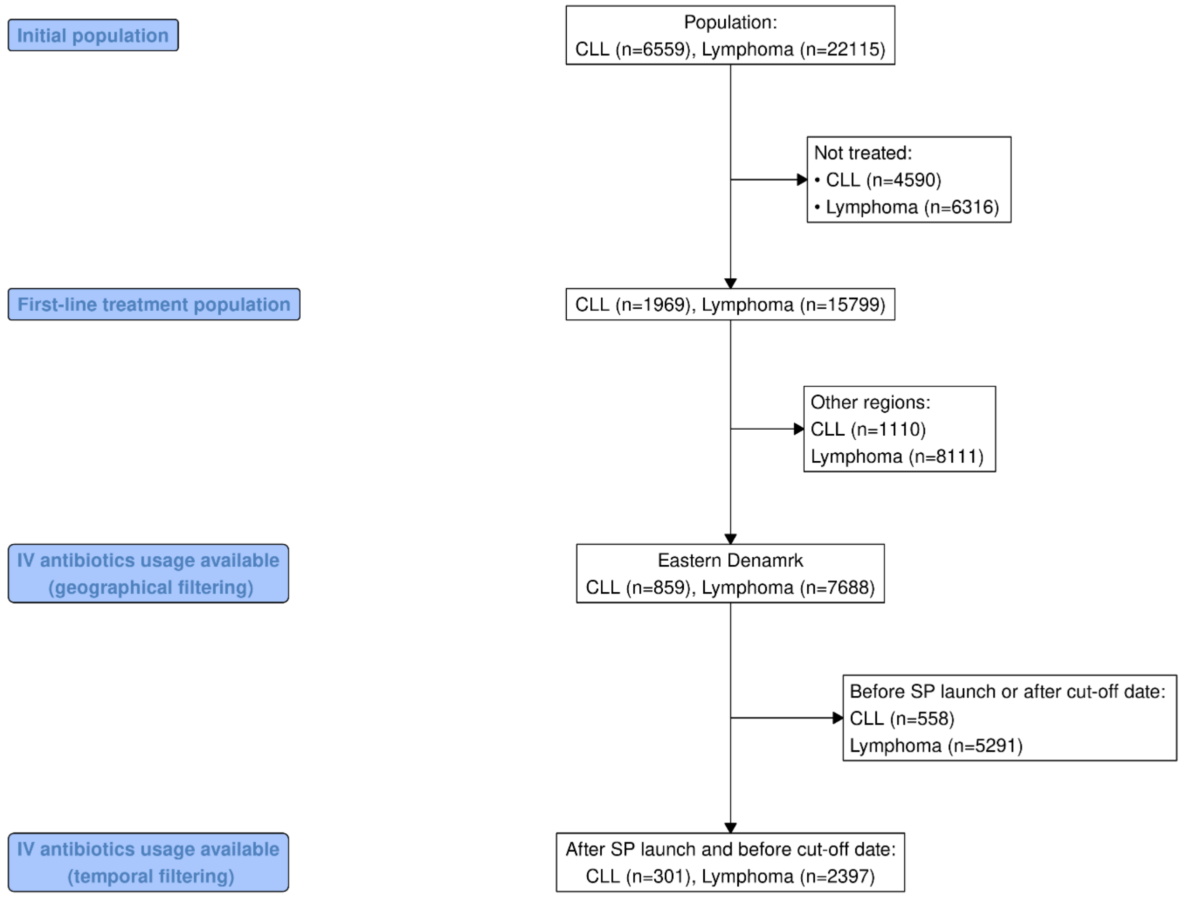

Figure S1: **Consort diagram illustrates the process of selecting patients from registries.** All the CLL and Lymphoma patients that received first-line treatment in eastern Denmark from the launch of SP have included in the final analysis.

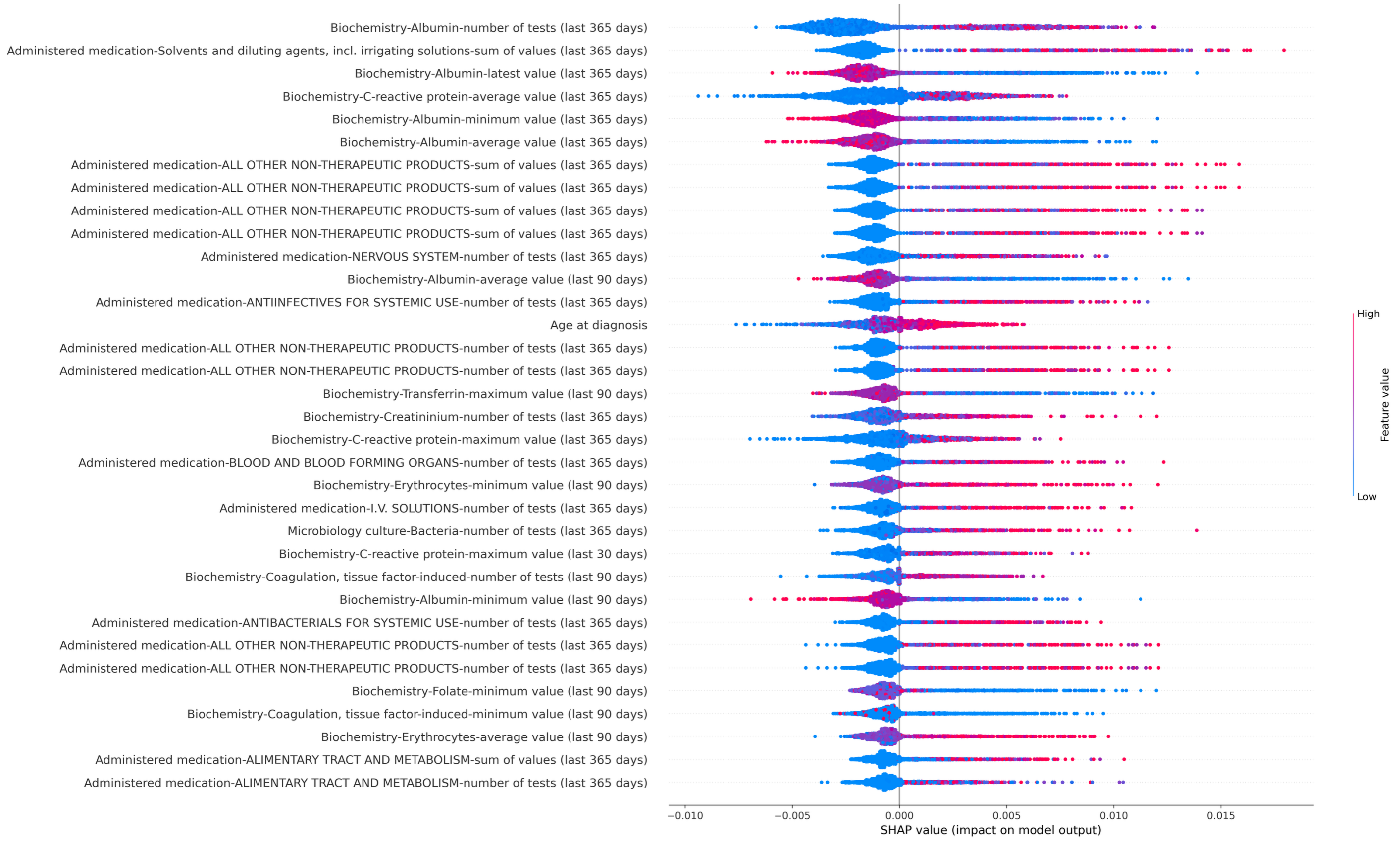

Figure S2 **Feature contributions of domain-specific model in predicting the risk of severe infection upon treatment estimated using SHAP.** The impact of a feature (average SHAP value) is shown on the x-axis and the top features are shown on the y-axis. The color represents the value of the feature from low to high. The most important features (average absolute SHAP value larger than 0.001) are displayed.

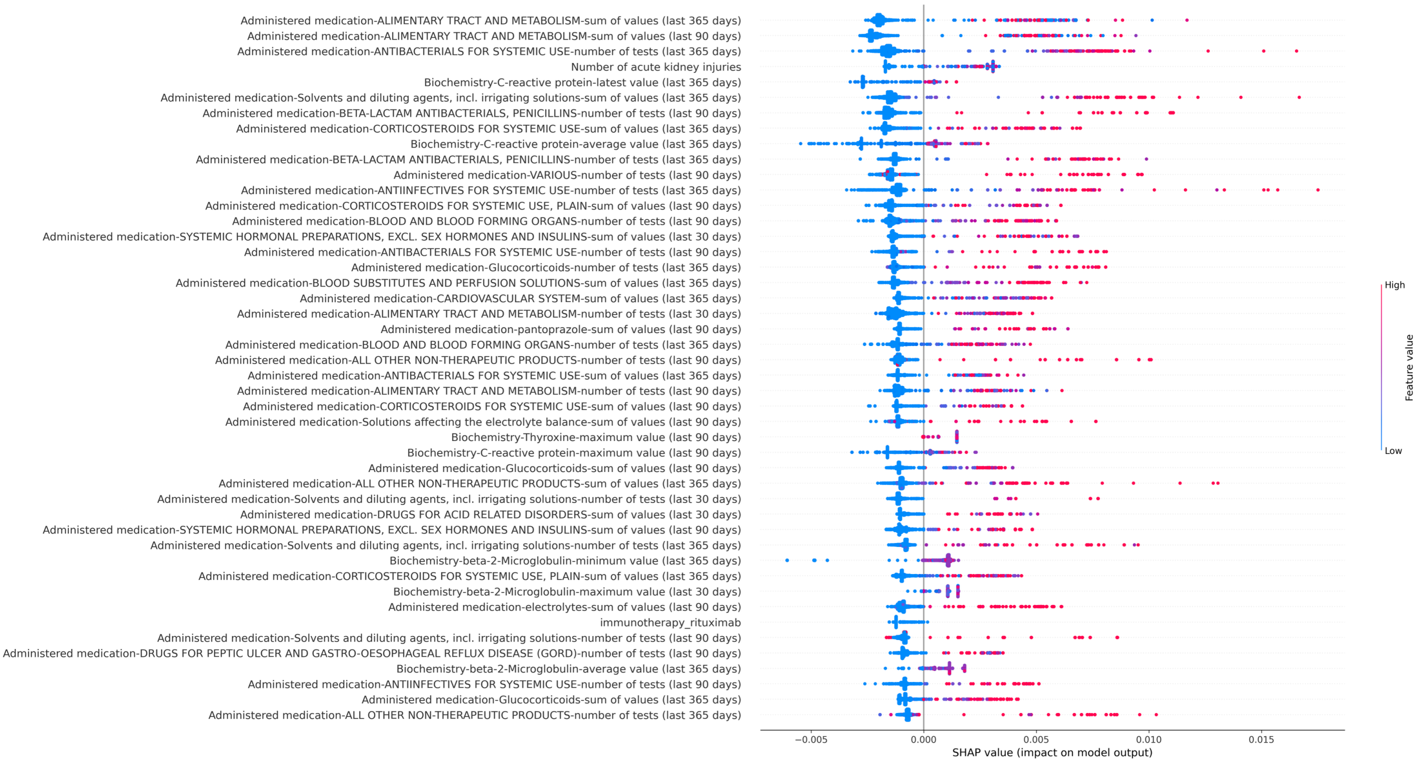

Figure S3 **Feature contributions of domain adaptation model in predicting the risk of severe infection upon treatment estimated using SHAP.** See Figure S2 legend for SHAP summary plot description.

Comparison with CLL-IPI

In the Results section of the paper, we defined IPI-based models as those trained on the individual components of the CLL-IPI score. As an alternative approach, we directly compared the CLL_{DA} model with the overall CLL-IPI risk score. To facilitate this comparison, we derived three binary risk classifiers from the four original CLL-IPI risk categories (low, intermediate, high, and very high) and evaluated their performance:

- Model 1: The low and intermediate levels were combined into a single low-risk category, and the high and very high levels were combined into a high-risk category. All patients were retained in this four-to-two-level grouping, and a binary classifier was constructed and tested on the full dataset.

- Model 2: The intermediate level was excluded; patients in the low category were labeled low risk, and those in the high or very high categories were labeled high risk.

- Model 3: A stricter grouping was applied: only the low category was considered low risk, and only the very high category was considered high risk.

Models 2 and 3 were necessarily evaluated on much smaller patient subsets because many cases fell into the excluded intermediate or high (but not very high) categories. Missing CLL-IPI values were imputed using the median of the observed scores.

The results showed that in the broadest grouping (Model 1, which included all patients), CLL_{DA} clearly outperformed both the standard CLL-IPI score and the CLLDS model (Table S5). In contrast, in the more extreme and smaller cohorts used for Models 2 and 3, CLL-IPI and CLL_{DS} achieved higher performance than CLL_{DA}, whose accuracy declined. The superior performance of CLL-IPI and CLL_{DS} in Models 2 and 3 is likely attributable to the fact that these models compare more sharply separated, extreme-risk groups that may be rare in the lymphoma dataset.

Table S5. The performance (MCC) of the DS and DA models, as well as a CLL-IPI–based prediction using three rules on CLL-IPI levels

	**Grouping of CLL-IPI Levels**		
**Models**	{L, I} < {H, VH} (n=271)	{L} < {H, VH} (n=95)	{L} < {VH} (n=57)
**CLL-IPI**	0.087	**0.303**	0.315
**CLL_{DS}**	0.290	0.229	**0.479**
**CLL_{DA}**	**0.339**	0.236	0.283
L: Low, I: Intermediate, H: High, VH: Very high			

We further evaluated the performance of the DS and DA models across the individual CLL-IPI risk categories. Patients with missing CLL-IPI values were analyzed as a separate group. Overall, both models achieved performance levels comparable to the MCC values observed in the full cohort, with the exception of the high-risk level (Table S6). Patients classified within the high CLL-IPI categories exhibited the lowest predictive performance, indicating the difficulty of the model in this group. The very high CLL-IPI category contained relatively few patients, and thus the elevated performance estimates in this subgroup should be interpreted with caution due to the increased likelihood of statistical noise (Table 1 of the manuscript).

Table S6. Performance (MCC) of CLL_{DS} and CLL_{DA} across CLL-IPI levels

		**CLL-IPI Levels**			
**Models**	Unknown	Low	Intermediate	High	Very high
**CLL_{DS}**	0.276	**0.305**	0.336	-0.039	**0.768**
**CLL_{DA}**	**0.366**	0.211	**0.415**	**0.128**	0.375

Feature Correlations

Given the use of multiple temporal window sizes, aggregation strategies, and probably the correlated nature of several variables, a certain degree of similarity among many of the features used in this experiment is expected. To assess this, we computed pairwise correlations among the most important features identified by the DS and DA models (Figure S4 and Figure S5). The resulting correlation matrices reveal a spectrum of relationships: while some features exhibit strong correlations, often because they are derived from the same underlying variable, others show little to no correlation. This pattern suggests that the models leverage complementary information, capturing predictive signals from distinct features depending on the characteristics of each patient. We also note that the correlation between features is an important design consideration in the deployment of CLL-TIM, as it provides redundancy that helps the model handle previously unseen missingness scenarios during deployment.[8]

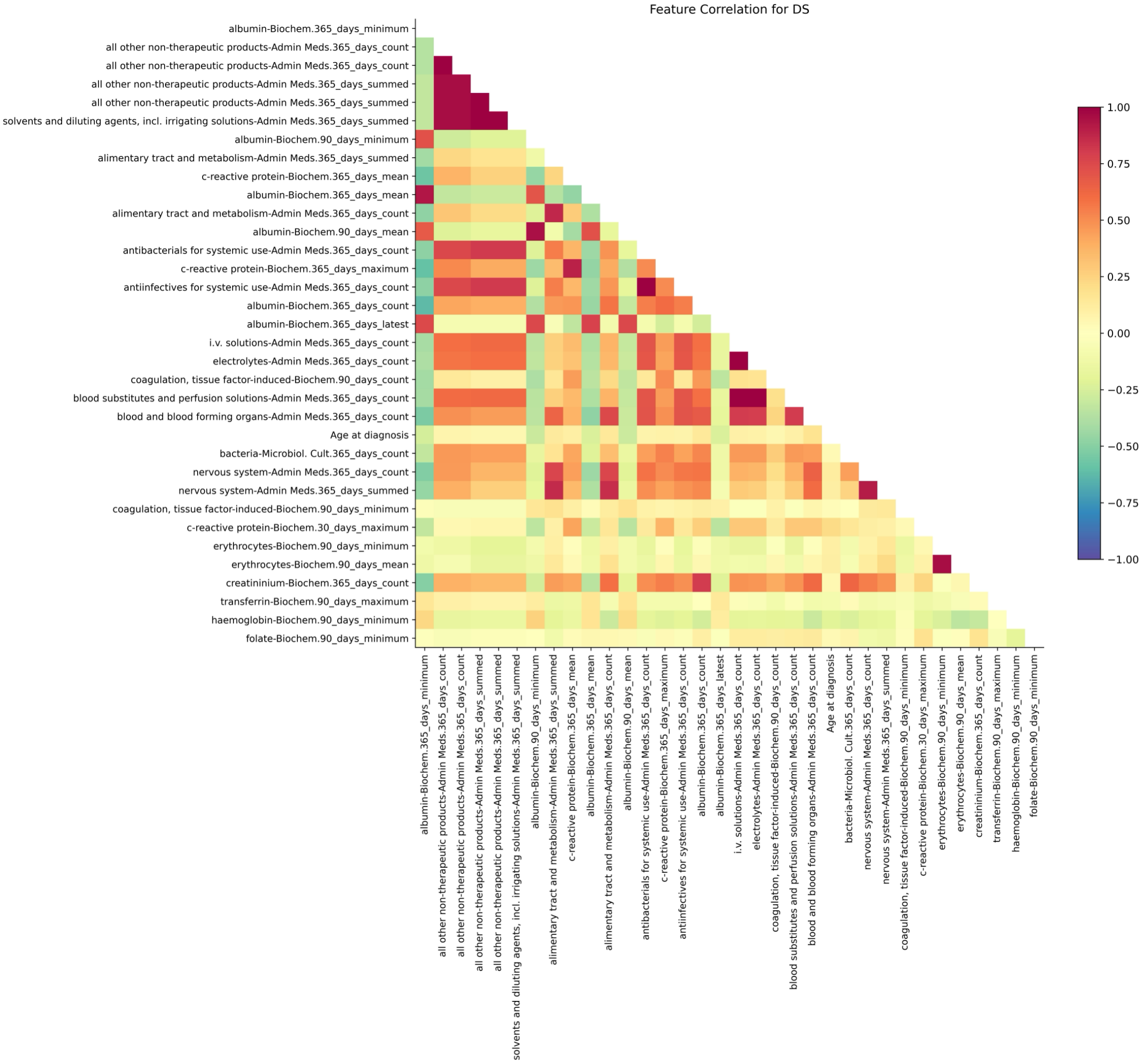

Figure S4. Correlation matrix of the most important features used by CLL_{DS} model

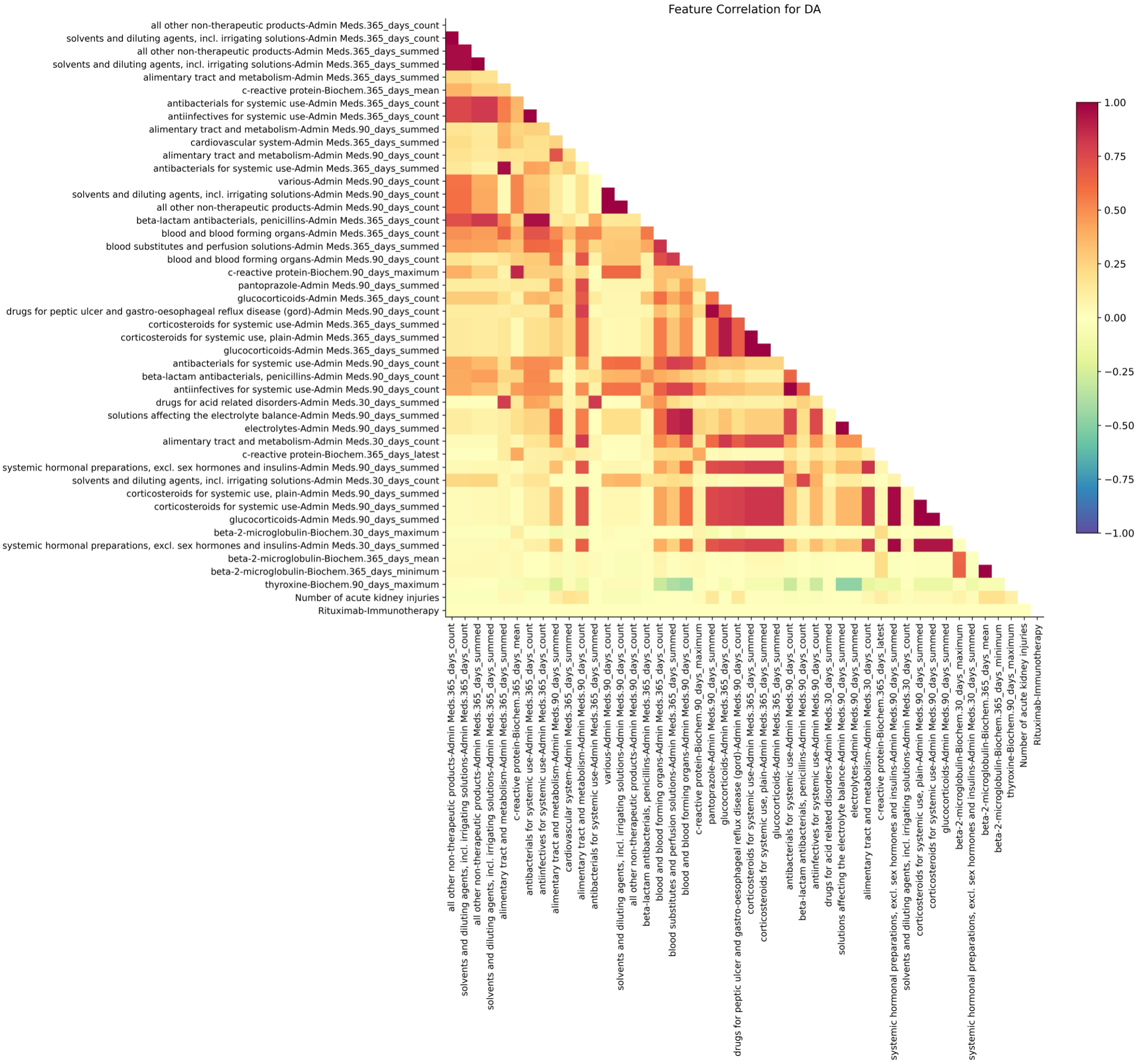

Figure S5. Correlation matrix of the most important features used by CLL$_{DA}$ model

Missingness

Table S7. Missing rate of the most important features used by CLL$_{DS}$ model

Feature name	missing rate (%)
albumin-Biochem.365_days_minimum	35.1
all other non-therapeutic products-Admin Meds.365_days_count	0
all other non-therapeutic products-Admin Meds.365_days_count	0
all other non-therapeutic products-Admin Meds.365_days_summed	0
all other non-therapeutic products-Admin Meds.365_days_summed	0
solvents and diluting agents, incl. irrigating solutions-Admin Meds.365_days_summed	0

albumin-Biochem.90_days_minimum	52
alimentary tract and metabolism-Admin Meds.365_days_summed	0
c-reactive protein-Biochem.365_days_mean	12.9
albumin-Biochem.365_days_mean	35.1
alimentary tract and metabolism-Admin Meds.365_days_count	0
albumin-Biochem.90_days_mean	52
antibacterials for systemic use-Admin Meds.365_days_count	0
c-reactive protein-Biochem.365_days_maximum	12.9
antiinfectives for systemic use-Admin Meds.365_days_count	0
albumin-Biochem.365_days_count	0
albumin-Biochem.365_days_latest	35.1
i.v. solutions-Admin Meds.365_days_count	0
electrolytes-Admin Meds.365_days_count	0
coagulation, tissue factor-induced-Biochem.90_days_count	0
blood substitutes and perfusion solutions-Admin Meds.365_days_count	0
blood and blood forming organs-Admin Meds.365_days_count	0
Age at diagnosis	0
bacteria-Microbiol. Cult.365_days_count	0
nervous system-Admin Meds.365_days_count	0
nervous system-Admin Meds.365_days_summed	0
coagulation, tissue factor-induced-Biochem.90_days_minimum	60.5
c-reactive protein-Biochem.30_days_maximum	25.1
erythrocytes-Biochem.90_days_minimum	53.9
erythrocytes-Biochem.90_days_mean	53.9
creatininium-Biochem.365_days_count	0
transferrin-Biochem.90_days_maximum	64.6
haemoglobin-Biochem.90_days_minimum	13.3
folate-Biochem.90_days_minimum	63.8

Table S8. Missing rate of the most important features used by CLL_{DA} model

**Feature name**	**missing rate (%)**
all other non-therapeutic products-Admin Meds.365_days_count	0
solvents and diluting agents, incl. irrigating solutions-Admin Meds.365_days_count	0
all other non-therapeutic products-Admin Meds.365_days_summed	0
solvents and diluting agents, incl. irrigating solutions-Admin Meds.365_days_summed	0
alimentary tract and metabolism-Admin Meds.365_days_summed	0
c-reactive protein-Biochem.365_days_mean	12.9
antibacterials for systemic use-Admin Meds.365_days_count	0
antiinfectives for systemic use-Admin Meds.365_days_count	0
alimentary tract and metabolism-Admin Meds.90_days_summed	0
cardiovascular system-Admin Meds.365_days_summed	0
alimentary tract and metabolism-Admin Meds.90_days_count	0

antibacterials for systemic use-Admin Meds.365_days_summed	0
various-Admin Meds.90_days_count	0
solvents and diluting agents, incl. irrigating solutions-Admin Meds.90_days_count	0
all other non-therapeutic products-Admin Meds.90_days_count	0
beta-lactam antibacterials, penicillins-Admin Meds.365_days_count	0
blood and blood forming organs-Admin Meds.365_days_count	0
blood substitutes and perfusion solutions-Admin Meds.365_days_summed	0
blood and blood forming organs-Admin Meds.90_days_count	0
c-reactive protein-Biochem.90_days_maximum	14
pantoprazole-Admin Meds.90_days_summed	0
glucocorticoids-Admin Meds.365_days_count	0
drugs for peptic ulcer and gastro-oesophageal reflux disease (gord)-Admin Meds.90_days_count	0
corticosteroids for systemic use-Admin Meds.365_days_summed	0
corticosteroids for systemic use, plain-Admin Meds.365_days_summed	0
glucocorticoids-Admin Meds.365_days_summed	0
antibacterials for systemic use-Admin Meds.90_days_count	0
beta-lactam antibacterials, penicillins-Admin Meds.90_days_count	0
antiinfectives for systemic use-Admin Meds.90_days_count	0
drugs for acid related disorders-Admin Meds.30_days_summed	0
solutions affecting the electrolyte balance-Admin Meds.90_days_summed	0
electrolytes-Admin Meds.90_days_summed	0
alimentary tract and metabolism-Admin Meds.30_days_count	0
c-reactive protein-Biochem.365_days_latest	12.9
systemic hormonal preparations, excl. sex hormones and insulins-Admin Meds.90_days_summed	0
solvents and diluting agents, incl. irrigating solutions-Admin Meds.30_days_count	0
corticosteroids for systemic use, plain-Admin Meds.90_days_summed	0
corticosteroids for systemic use-Admin Meds.90_days_summed	0
glucocorticoids-Admin Meds.90_days_summed	0
beta-2-microglobulin-Biochem.30_days_maximum	88.2
systemic hormonal preparations, excl. sex hormones and insulins-Admin Meds.30_days_summed	0
beta-2-microglobulin-Biochem.365_days_mean	66.1
beta-2-microglobulin-Biochem.365_days_minimum	66.1
thyroxine-Biochem.90_days_maximum	90.4
Number of acute kidney injuries	0
Rituximab-Immunotherapy	0

As noted in the main discussion regarding feature availability, the missing-data rate is higher among patients without a history of hospitalization compared with those who have been hospitalized (Table S9). This pattern is consistent in both the CLL and lymphoma cohorts. In contrast, the relationship between infection status and missingness differs between the two diseases. Among CLL patients, those who experienced an infection exhibited a lower missing-data rate, likely reflecting a greater number of prior medical encounters and, consequently, more comprehensive clinical records. For lymphoma patients, however, a slight increase in missingness was observed among those with infections. This may

indicate that infection events in lymphoma are documented differently or are associated with distinct clinical pathways compared with CLL.

Table S9. Percentages of missing data for CLL and lymphoma patients, stratified by infection and hospitalization status

	**CLL**		**Lymphoma**	
	**Infection**	**No infection**	**Infection**	**No infection**
**Hospitalized**	13.6	15.7	10.3	8.5
**Not hospitalized**	16.1	22.4	12.1	11.5

We also evaluated data completeness at the patient level, particularly among those without a history of hospitalization (Table S10). While enforcing data availability improved performance for the CLL_{DA} model, it resulted in poorer performance for the CLL_{DS} model. In the DA strategy, requiring complete data caused the retained CLL patients to resemble the lymphoma cohort more closely in terms of feature distributions, which may explain the observed performance gain. In contrast, in the DS strategy, some missingness patterns appear to be informative rather than random. Consequently, enforcing completeness likely removed patients for whom the pattern of missing values itself carried predictive information about infection risk. In other words, requiring complete data may discard cases where missingness reflects meaningful clinical processes rather than random data absence, ultimately reducing predictive performance.

Table S10. Performance analysis of non-hospitalized patients across varying levels of data completeness

**Completeness**	**CLL_{DS}**	**CLL_{DA}**
**90%**	-0.115	**0.299**
**80%**	0.046	0.203
**70%**	0.087	0.203
**No exclusion**	0.075	0.113

Implications

This study showcases the potential of utilizing an ML-based algorithm to stratify patients with CLL according to their risk of experiencing severe infections across different treatment regimens. Given that infections are the leading cause of morbidity and mortality in CLL, this represents an important advancement towards developing individualized therapies based on the risk of adverse events. Future research should focus on a comprehensive risk assessment that accounts for multiple adverse outcomes including bleeding and cardiac events,[9] which also put patients with CLL at risk upon treatment. Upon internal and external validation of such a comprehensive model, the road to clinical deployment may take the same path as built for CLL-TIM.[8] This study also demonstrates the potential of using domain adaption to improve predictions for a given diseases by training on data from neighboring diseases. Building upon these findings, we

emphasize the importance of merging data from different data sources and disease entities to improve the performance for a given target disease.

References

1. AF G, R E, AG N, T F, RW T. Existing data sources for clinical epidemiology: The clinical laboratory information system (LABKA) research database at Aarhus University, Denmark. *Clin Epidemiol*. 2011;3:133. doi:10.2147/CLEP.S17901

2. Persimune. Danish National Foundation for Research's (DNRF) centre of excellence (COE) for personalised medicine of infectious complications in immune deficiency (PERSIMUNE). https://www.persimune.dk/

3. Da Cunha-Bang C, Geisler CH, Enggaard L, et al. The Danish National Chronic Lymphocytic Leukemia Registry. *Clin Epidemiol*. 2016;8:561-565. doi:10.2147/CLEP.S99486

4. Johannesdottir SA, Horváth-Puhó E, Ehrenstein V, Schmidt M, Pedersen L, Sørensen HT. Existing data sources for clinical epidemiology: The Danish National Database of Reimbursed Prescriptions. *Clin Epidemiol*. 2012;4(1):303-313. doi:10.2147/CLEP.S37587

5. Schmidt M, Schmidt SAJ, Sandegaard JL, Ehrenstein V, Pedersen L, Sørensen HT. The Danish National Patient Registry: a review of content, data quality, and research potential. *Clin Epidemiol*. 2015;7:449. doi:10.2147/CLEP.S91125

6. Arboe B, Josefsson P, Jørgensen J, et al. Danish National Lymphoma Registry. *Clin Epidemiol*. 2016;8:577-581. doi:10.2147/CLEP.S99470

7. Petersen CT, Jensen KJ, Rosenzweig M, von Osmanski BI, Ankarfeldt MZ, Petersen J. Mapping Outcomes and Registries Used in Current Danish Pharmacoepidemiological Research. *Clin Epidemiol*. 2022;14:521-542. doi:10.2147/CLEP.S341480

8. Agius R, Riis-Jensen AC, Wimmer B, et al. Deployment and validation of the CLL treatment infection model adjoined to an EHR system. *npj Digit Med 2024 71*. 2024;7(1):1-12. doi:10.1038/s41746-024-01132-6

9. Parviz M, Agius R, Rotbain EC, Vainer N, Aarup K, Niemann CU. Identifying CLL patients at high risk of atrial fibrillation on treatment using machine learning. *Leuk Lymphoma*. 2024;65(4):449-459. doi:10.1080/10428194.2023.2299737
